# miR-22-3p as a potential biomarker for coronary artery disease based on integrated bioinformatics analysis

**DOI:** 10.3389/fgene.2022.936937

**Published:** 2022-08-29

**Authors:** Minghua Zhang, Yan Hu, Haoda Li, Xiaozi Guo, Junhui Zhong, Sha He

**Affiliations:** ^1^ Department of Cardiovascular Medicine, Key Laboratory of Biological Targeting Diagnosis, Therapy and Rehabilitation of Guangdong Higher Education Institutes, The Fifth Affiliated Hospital, Guangzhou Medical University, Guangzhou, China; ^2^ Nursing Department, Key Laboratory of Biological Targeting Diagnosis, Therapy and Rehabilitation of Guangdong Higher Education Institutes, The Fifth Affiliated Hospital, Guangzhou Medical University, Guangzhou, China; ^3^ Key Laboratory of Biological Targeting Diagnosis, Therapy and Rehabilitation of Guangdong Higher Education Institutes, The Fifth Affiliated Hospital, Guangzhou Medical University, Guangzhou, China

**Keywords:** microRNA, miR-22-3p, coronary artery disease, gene expression, blood

## Abstract

**Background:** Coronary artery disease (CAD) is a common cardiovascular disease that has attracted attention worldwide due to its high morbidity and mortality. Recent studies have shown that abnormal microRNA (miRNA) expression is effective in CAD diagnoses and processes. However, the potential relationship between miRNAs and CAD remains unclear.

**Methods:** Microarray datasets GSE105449 and GSE28858 were downloaded directly from the Gene Expression Omnibus (GEO) to identify miRNAs involved in CAD. Target gene prediction and enrichment analyses were performed using Gene Ontology (GO) and Kyoto Encyclopedia of Genes and Genomes (KEGG).

**Results:** There were nine differentially expressed miRNAs in CAD patients compared to the controls. A total of 352 genes were predicted and subjected to GO analysis, which showed that differentially expressed genes (DEGs) were mainly associated with axon guidance, neuron projection guidance, neuron-to-neuron synapses, and postsynaptic density. According to the KEGG pathway analysis, the most enriched pathways were those involved in transcriptional misregulation in cancer, growth hormone synthesis, secretion and action, endocrine resistance, axon guidance, and Cushing syndrome. Pathway analysis was mainly involved in the HIPPO and prion disease signaling pathways. Furthermore, a competing endogenous RNA (ceRNA) interaction network centered on miR-22-3p revealed eight related transcription factors in the cardiovascular system. The receiver operating characteristic (ROC) curve analysis suggested that miR-22-3p may be a better CAD predictor.

**Conclusion:** The results indicate that miR-22-3p may function in pathophysiological CAD processes. Our study potentiates miR-22-3p as a specific biomarker for diagnosing CAD.

## 1 Introduction

Coronary artery disease (CAD) is a cardiovascular disease with a high global morbidity and mortality rate. Globally, it has caused serious social and economic burdens and has become a major health problem (Karakas et al., 2017). Although advancements in medical technology have continuously improved CAD treatment methods, including double-chain antiplatelet, enhanced low-density lipoprotein cholesterol reduction, and coronary stent implantation, there is still a lack of one or more biomarkers with high specificity and sensitivity for early diagnosis of CAD (Thomas and Lip, 2017). Therefore, it is of great significance to find biomarkers for early diagnosis of CAD through non-invasive and convenient methods. MicroRNAs (miRNAs) are a group of naturally occurring non-coding small RNAs, 21–25 nucleotides long, which regulate the expression of target genes by specifically inhibiting or degrading the translation of target mRNAs. It has been recently discovered that detecting non-invasive biomarker miRNAs (such as miR-15b-5p, miR-29c-3p, and miR-199a-3p) can provide a powerful means for predicting and diagnosing CAD (Su et al., 2020), helping clinicians provide the best prevention and treatment plans for CAD patients as soon as possible. Biomarker miRNA detection can greatly improve the overall prognosis of CAD patients.

Selective coronary angiography (CAG) is the current gold standard for diagnosing CAD. However, this surgical method is invasive, cumbersome, and expensive. It is mainly used in the late stages of the disease when multiple blood vessels are affected or arteries are seriously stenosed (Su et al., 2020). At present, the most widely used traditional biomarkers for diagnosing coronary heart disease, such as creatine kinase MB, B-type brain natriuretic peptide precursors, and high-sensitivity troponin T/I, are affected by age, genetic background, heart-related diseases, drugs, and lifestyle and cannot be used for early diagnosis of acute myocardial infarction nor can they predict the future complications of coronary heart disease (Raggi et al., 2018; Tanase et al., 2021).

In recent years, deep sequencing and microarrays have effectively detected complex networks in the atherosclerosis process. They can be used as biomarkers in CAD patient diagnosis and prognosis (Tan et al., 2017). The combination of microarray technology and bioinformatics analysis methods can comprehensively analyze the early-to-late module of gene expression changes in atherosclerosis development (Gu et al., 2021). Gene Expression Omnibus (GEO) is a public database containing numerous human gene profiles for multiple diseases. It is commonly used to screen for differentially expressed genes (DEGs) and construct regulatory networks of gene interactions (Meng et al., 2021). Bioinformatics analysis methods can provide effective biomarker candidates for clinical trials and clinical practice (Cheng et al., 2019). Lin et al. analyzed differentially expressed long non-coding RNAs (lncRNAs) (DELs) and differentially expressed coding genes in vascular smooth muscle cells (human aortic smooth muscle cells (HASMCs)) and found that hypoxia-inducible factor-1 alpha (HIF-1α) antisense RNA2 partially inhibited HASMC proliferation through the miR-30e-5p/ccnd2 axis (Lin et al., 2021). Another bioinformatics analysis study identified miRNA-376a-3p as a novel biomarker in CAD patients (Du et al., 2020).

In this study, we downloaded the original data of CAD and healthy control samples for microarray analysis from the GEO database. We used bioinformatics analysis methods to potentiate effective candidate biomarkers and key factors for early CAD screening and prognosis determination.

## 2 Materials and methods

### 2.1 Data acquisition

The GEO query package (Kok et al., 2015) of RStudio (version 3.6.5, http://r-project.org/) was used to download the coronary heart disease expression profile datasets GSE105449 (de Ronde et al., 2017) and GSE28858 (Sondermeijer et al., 2011) using *Homo sapiens* samples. The GSE105449 dataset was used as the test set. The platform was based on GPL22949, including 38 blood samples from patients with cardiovascular disease, 25 blood specimens from healthy individuals taking cardiovascular disease drugs (control 1), and 42 blood samples from healthy individuals not taking cardiovascular disease drugs (control 2). The GSE28858 dataset was a GPL8179-based validation set and included 12 blood samples from patients with cardiovascular disease and 12 blood samples from normal subjects (Table 1). We processed the raw data from the GSE105449 and GSE28858 datasets *via* the “affy” package (Gautier et al., 2004) and background-corrected and normalized the data. The gene expression matrices of the two datasets were obtained separately. The effect of inter-sample correction was demonstrated by plotting BOX and principal components analysis (PCA) with the “ggplot2” package (Wilkinson, 2011).

**TABLE 1:** Information of two datasets.

Dataset	Platforms	Organism	Source	CVD	Control
GSE105449	GPL22949	Homo sapiens	monocyte	38	25 (1)
					42 (2)
GSE28858	GPL8179	Homo sapiens	platelets	12	12

CVD, Cardiovascular disease.

(1), Monocytes CTRL after medication

(2), Monocytes CTRL without medication

### 2.2 Boxplot analysis of the hsa-miR-22-3p gene

The hsa-miR-22-3p gene expression distribution values under different groups in the GSE105449 and GSE28858 datasets were visualized using a boxplot.

### 2.3 Receiver operating characteristic (ROC) analysis of the hsa-miR-22-3p gene

The GSE105449 and GSE28858 datasets were analyzed using the pROC package to construct a hsa-miR-22-3p molecular expression and prediction outcome model using the area under the ROC curve (AUC) to analyze prediction efficacy. The AUC value is the total area covered by the ROC curve. A larger AUC value indicated a better classifier.

### 2.4 DEG screening

We screened the GSE105449 dataset for DEGs by downloading the “limma” package (Ritchie et al., 2015), and heatmaps were drawn using the pheatmap package (Kolde, 2019) to show the differential distribution of sample DEGs. The DEG volcano maps were illustrated using the “ggplot2” package to present the differential expression of DEGs.

### 2.5 miRNA and mRNA network analysis and functional analysis

The miRWalk database (Dweep and Gretz, 2015) predicted nine DEGs, and the DEGs were demonstrated using Target Scan’s gene list. DEG Gene Ontology (GO) and Kyoto Encyclopedia of Genes and Genomes (KEGG) pathway enrichment analyses were performed using the clusterProfiler package (Yu et al., 2012), and *p* < 0.05 was considered statistically significant. The DIANA TOOLS database showed pathway maps for pathways enriched in KEGGs (Vlachos et al., 2015).

### 2.6 DEGs and lncRNA network analysis

Possible lncRNAs for DEGs were predicted from the starBase v2.0 database (Ma et al., 2015), screening experimental grade >1. DEGs correlated with miRNAs, and lncRNAs were visualized using Cytoscape.

### 2.7 DEGs and transcription factor network analysis

We predicted the possible transcription factors of DEGs from the TransmiRv2.0 database (http://www.cuilab.cn/transmir) and visualized DEGs with transcription factors and the tissue association results using Cytoscape.

### 2.8 Statistical analysis

All data processing and analyses were performed using R software (version 4.0.2). For comparisons between two groups of continuous variables that conformed to a normal distribution, an independent Student’s *t*-test was used to determine whether the variables were statistically different. For comparisons between non-normally distributed variables, a Mann–Whitney U-test (i.e., Wilcoxon rank-sum test) was used to analyze whether there was a statistical difference. Statistical significance between the two sets of categorical variables was compared and analyzed using a chi-square test or Fisher’s exact probability test. The correlation coefficients between different genes were determined using Pearson’s correlation analysis. All statistical *p*-values were two-sided, and statistical significance was set at *p* < 0.05.

## 3 Results

### 3.1 DEG screening

**FIGURE 1:**
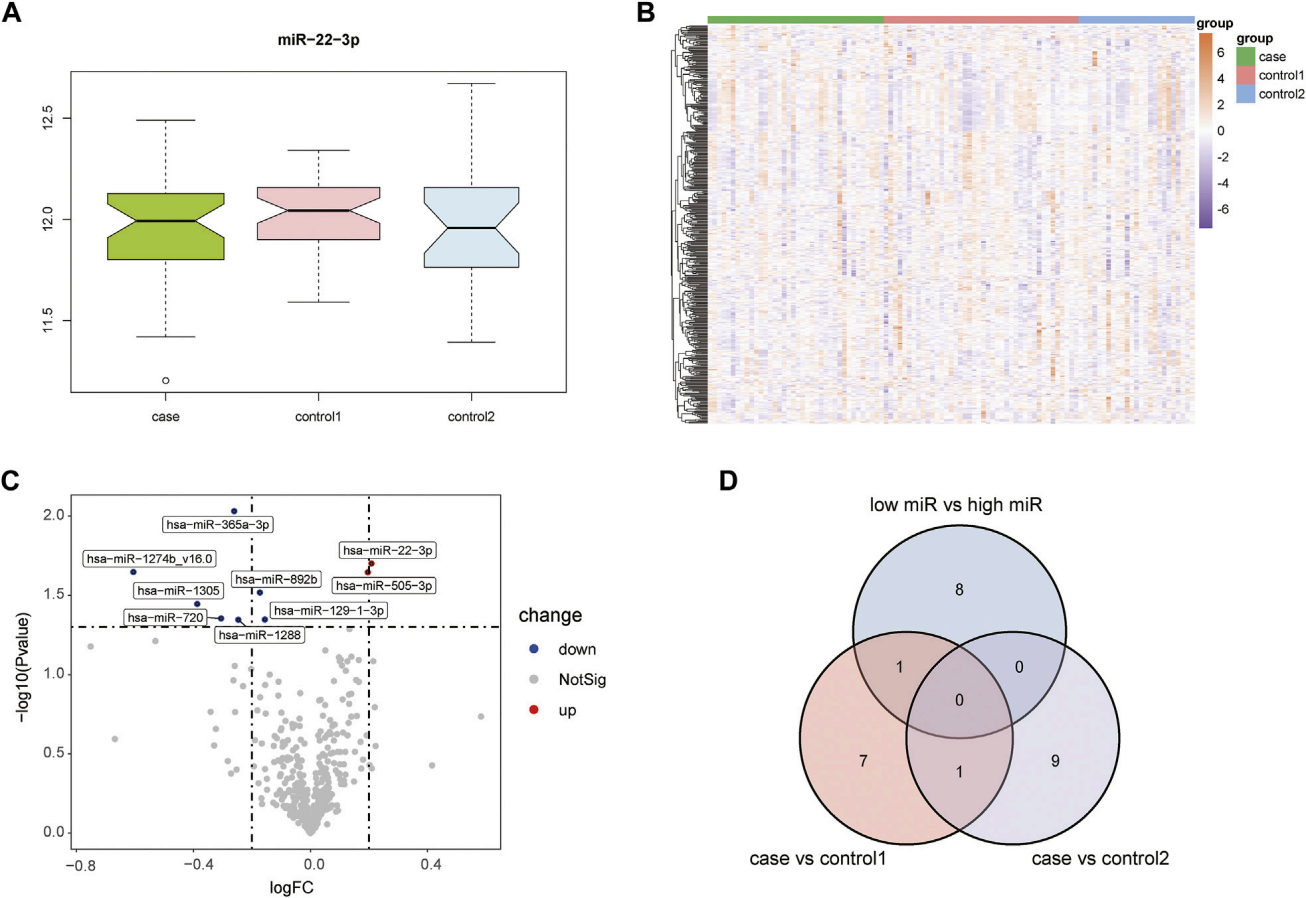
Acquisition of DEGs from the GSE105449 dataset. **(A)** Boxplot showing miR-22-3p expression. **(B)** Heatmap showing the mRNA expression profiles of case samples, control 1 and control 2. **(C)** Volcano map of DEGs. Red, green, and gray represent upregulated differential genes, downregulated differential genes, and no differential genes, respectively. **(D)** Venn diagram of overlapping DEGs from the intersection of two independent groups.

Supplementary Figure S1 shows the boxplots illustrating GSE105449 and GSE28858, and Figure S2 displays the PCA plots. Their findings showed that the samples of the two groups clustered more obviously after preprocessing, indicating that the samples were obtained from reliable sources. The GSE105449 dataset consisted of the case, control 1, and control 2 groups. The expression of miR-22-3p in the three groups of samples was detected and plotted with a box diagram (Figure 1A) and heat map (Figure 1B). In the case group, the disease groups were divided into high- and low-expression groups based on the median miR-22-3p value, and volcano plot analysis was performed to obtain nine DEGs: hsa-miR-365a-3p, hsa-miR-22-3p, hsa-miR-1274b_v16.0, hsa-miR-505-3p, hsa-miR-892b, hsa-miR-1305, hsa-miR-720, hsa-miR-129-1-3p, and hsa-miR-1288 (Figure 1C). The case group versus control 1 group and case group versus control 2 group DEGs were obtained using the “limma” package, and a Venn diagram displays the three-part genes (Figure 1D).

**FIGURE 2:**
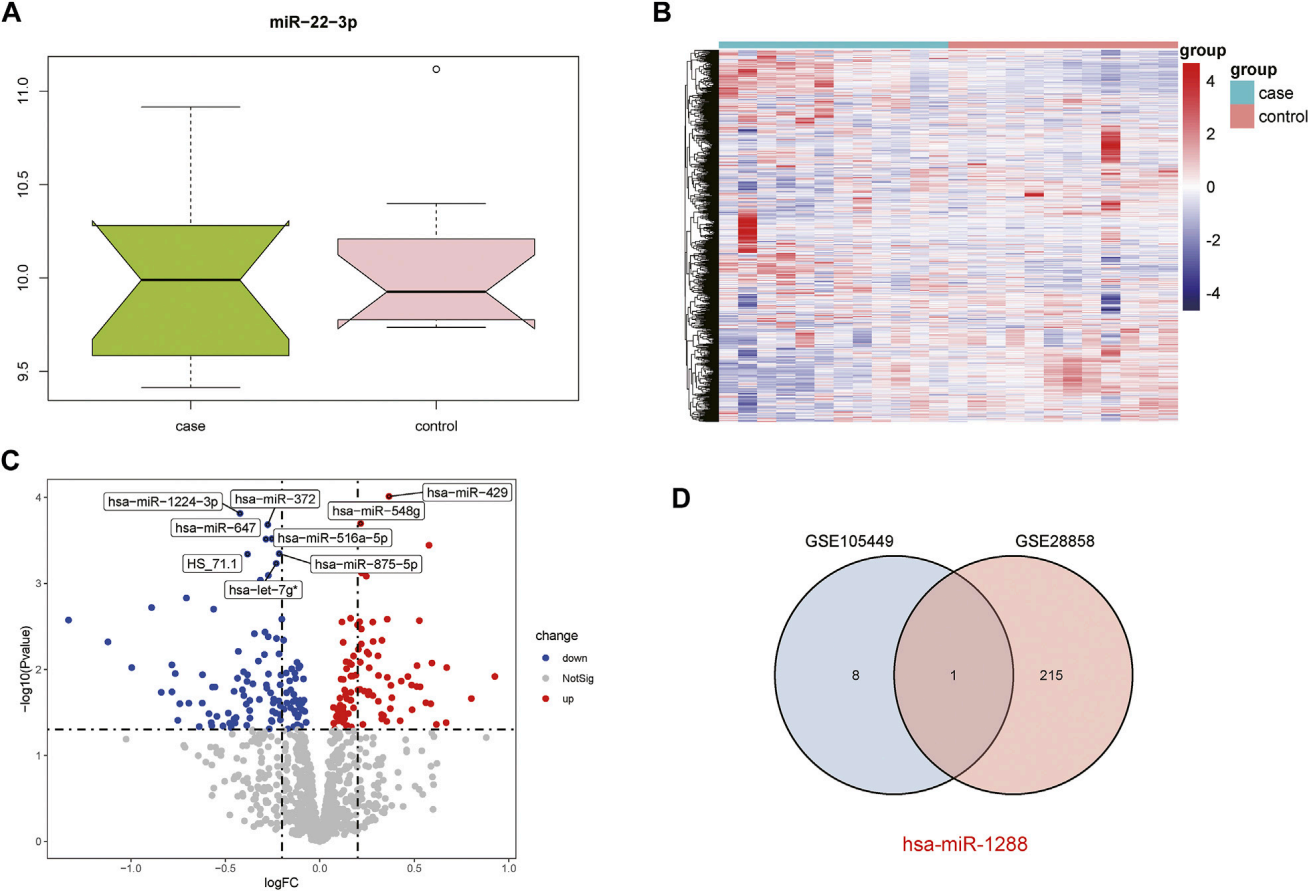
Acquisition of DEGs from the GSE28858 dataset. **(A)** Boxplot showing miR-22-3p expression. **(B)** Heatmap showing the mRNA expression profiles of case samples, control 1 and control 2. **(C)** DEG volcano map. Red, green, and gray represent upregulated differential genes, downregulated differential genes, and no differential genes, respectively. **(D)** Venn diagram of overlapping DEGs from the intersection of two independent datasets GSE105449 and GSE28858.

The GSE28858 dataset was used as the validation set and included the case and control groups. Hsa-miR-22-3p expression in the samples of both groups was extracted, as the boxplot (Figure 2A) and heatmap (Figure 2B) show. In the case group, the median hsa-miR-22-3p value was used to divide the high- and low-expression groups, and volcano plot analysis was performed to obtain 216 DEGs (Figure 2C). The Venn diagrams of the common differentially expressed miRNAs in both datasets detail this (Figure 2D).

### 3.2 Correlation analysis of DEGs

**FIGURE 3:**
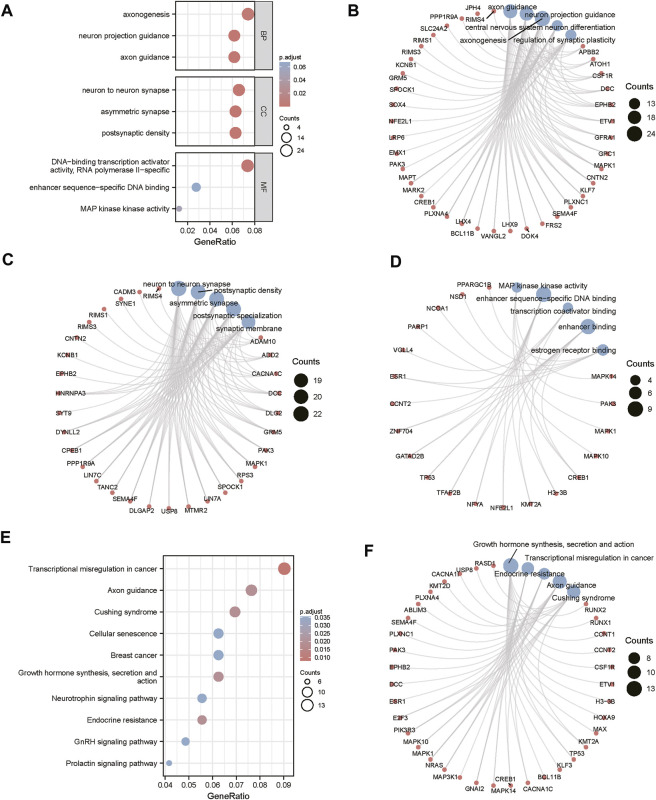
Functional correlation analysis of DEGs. **(A)** Functional enrichment analysis of the overall biology of GO. The X horizontal axis indicates the proportion of DEGs enriched in the GO terms, and the color of the dots shows the significant *p*-value: the redder the color, the smaller the corrected *p*-value; the bluer the color, the larger the corrected *p*-value. The size of the dots represents the number of enriched genes. **(B)** BP functional enrichment analysis; the color of the dots indicates the |logFC| of the genes. **(C)** CC functional enrichment analysis. **(D)** MF functional enrichment analysis. **(E)** KEGG pathway enrichment analysis. **(F)** Top five KEGG-enriched signaling pathways and related genes.

**FIGURE 4:**
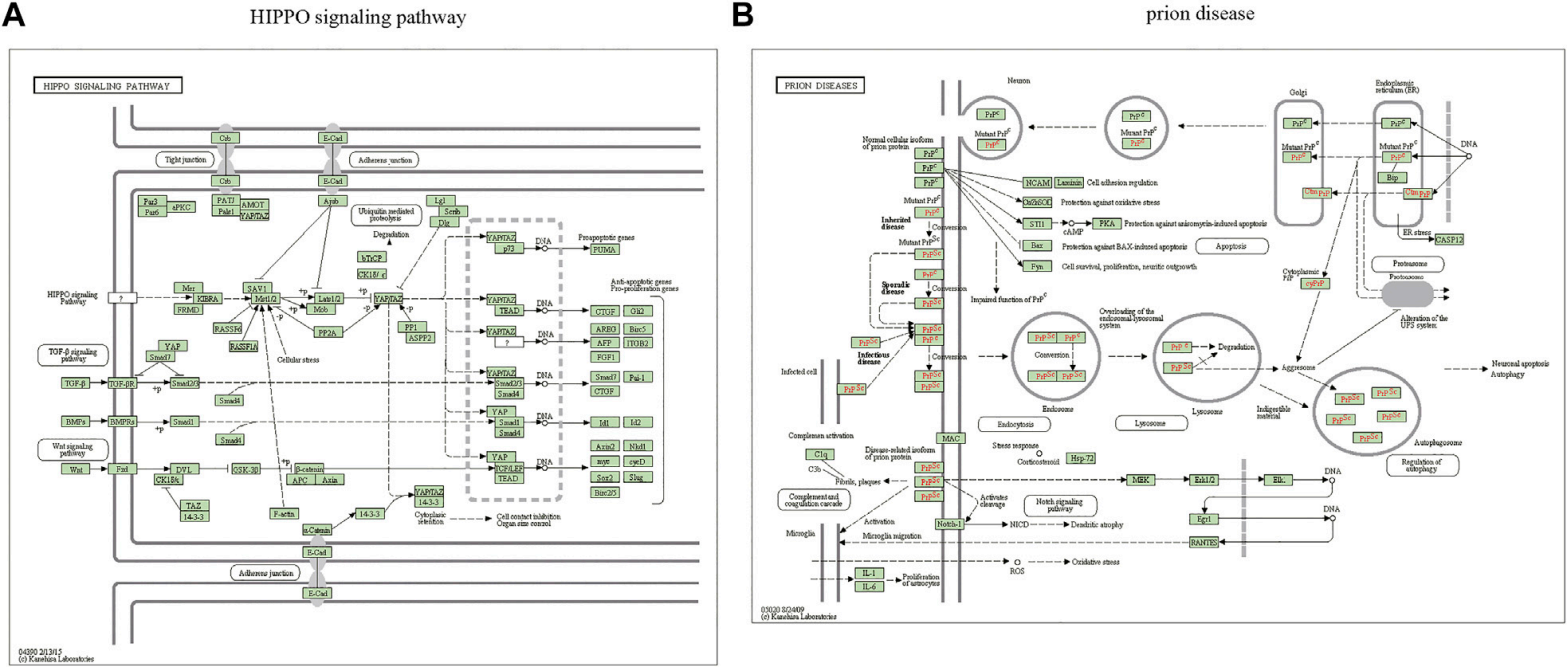
Pathview analysis of DEGs. **(A)** Pathview pathway map of the HIPPO signaling pathway. **(B)** Prion disease pathway map. Colors represent the location of DEGs in this pathway, and red represents upregulated genes.

The miRWalk website was used for DEG target gene prediction, and three DEGs (hsa-miR-22-3p, hsa-miR-129-1-3p, and hsa-miR-365a-3p) were included, with a validation level of 352 target genes predicted for enrichment analysis. GO analysis showed that the DEGs were mainly associated with axon guidance, neuron projection guidance, neuron-to-neuron synapses, and postsynaptic density (Figures 3A–D). Supplementary Table S1 details the results. The KEGG analysis (Figures 3E,F) revealed that the pathways enriched by DEGs mainly included transcriptional misregulation in cancer, growth hormone synthesis, secretion and action, endocrine resistance, axon guidance, and Cushing syndrome. Pathview-enriched pathways mainly involved the HIPPO signaling and prion disease pathways (Figures 4A,B).

### 3.3 Analysis of the competing endogenous RNA interaction network

**FIGURE 5:**
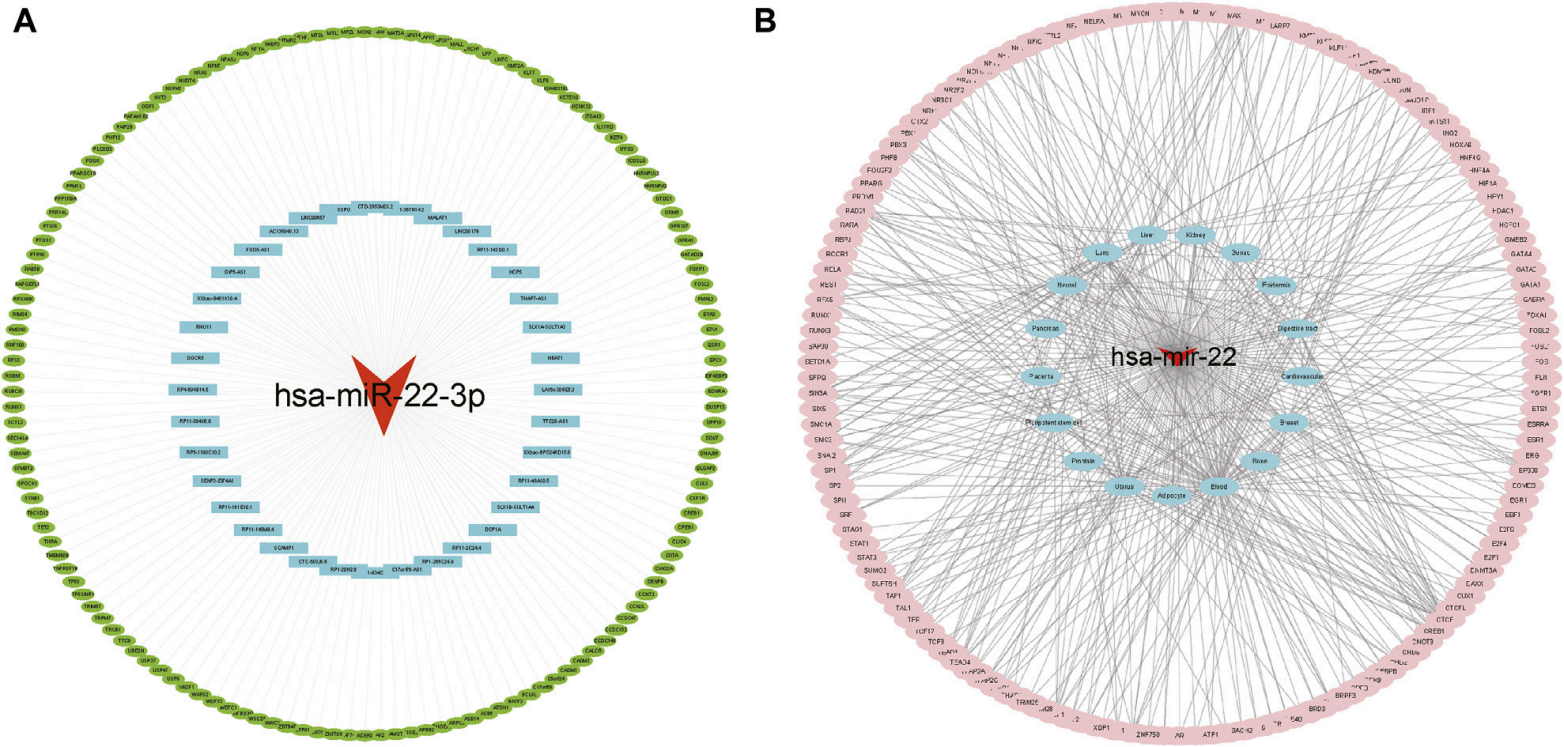
Interaction network analysis. **(A)** ceRNA network analysis. The red triangle represents miRNA, blue represents lncRNA, and green represents target genes. **(B)** Target gene-transcription factor analysis. Red represents miRNA genes, blue represents relevant disease tissue, and pink represents transcription factors.

The ceRNA interaction network of mRNA–miRNA-long intergenic non-coding RNA (lincRNA) was constructed using miR-22-3p as the center. As Figure 5A shows, 37 possible lncRNAs for DEGs were predicted by Starbase V2.0, and 142 possible lncRNAs for DEGs were predicted by the miRWalk database. Potential transcription factors for DEGs were predicted using the TransmiR v2.0 database (Figure 5B) for various tissues, such as the heart, kidney, and liver. A total of eight associated transcription factors, CCCTC-binding factor (CTCF), JUN, JUND, NFATC1, NFE2L2, RAD21, RELA, and TAL1, were identified in the cardiovascular system.

### 3.4 ROC curve analysis

**FIGURE 6:**
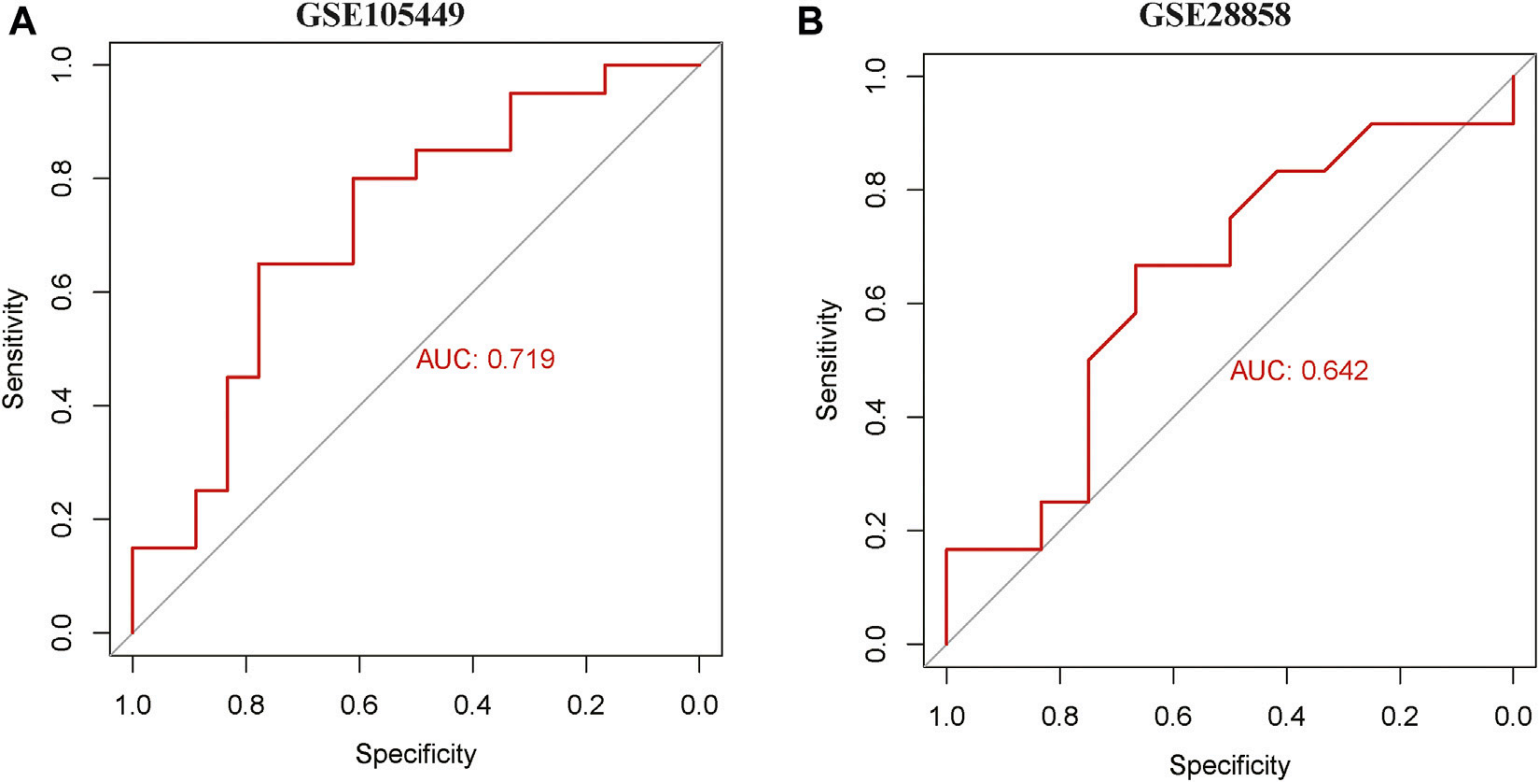
ROC curve analysis. **(A)** ROC curve of GSE105449. **(B)** ROC curve of GSE28858.

ROC curves of the GSE105449 and GSE28858 datasets were constructed with miR-22-3p as the center. The area under the AUC curve of GSE105449 was 0.719 (Figure 6A), whereas the area under the AUC curve of the GSE28858 validation set was 0.642 (Figure 6B).

## 4 Discussion

Numerous studies have found that miRNAs play a large role in cell differentiation, biological development, and disease development. Evidence indicated that circulating miRNAs are crucial in CAD progression (Mayr et al., 2021). However, there is no consensus regarding which miRNAs are clinically relevant for cardiovascular disease expression. miR-22-3p was first discovered as a miRNA with antitumor properties (Pandey and Picard, 2009). Previous studies have reported that miR-22-3p is abundantly expressed in the heart (Hu et al., 2012), where it is vital in vascular remodeling (Zheng and Xu, 2014) and cardiac hypertrophy (Gurha et al., 2012a; Huang et al., 2013). Our previous research confirmed that miR-22-3p directly targeting the transcription factor specificity protein 1 (Sp1) suppresses vascular smooth muscle cell proliferation and migration and vascular neointima formation (Zhang et al., 2020). Zeng et al. (2021) reported that circular RNA circMAP3K5 acts as a miRNA-22-3p sponge and the circMAP3K5/miR-22-3p/TET2 axis may be a potential target for endothelial proliferation-related diseases, including revascularization and atherosclerosis. Recent studies have reported dysregulation of signal transducer and activator of transcription 1 (STAT1), miR-150, miR-223, miR-21, and miR-25 in the peripheral blood mononuclear cells (PBMCs) of patients with definite CAD (Nariman-Saleh-Fam et al., 2019; Saadatian et al., 2019). Another study suggested that miR-22-3p was downregulated in CAD patients and promoted CAD progression by targeting the inflammatory response-related factor monocyte chemoattractant protein-1 (MCP-1) (Chen et al., 2016). The present studies on miRNAs are controversial and inconsistent, and the mechanisms and potential significance of miRNAs regulating CAD-associated gene levels remain unclear (Zhelankin et al., 2021).

In this study, we identified potential miRNAs for the diagnosis and treatment of coronary heart disease based on two GEO datasets by analyzing differences in the expression of critical genes. However, the present study observed that PBMC miR-22-3p was increased in CAD patients compared to healthy control individuals (control 1 and control 2), suggesting that miR-22-3p upregulation might be crucial in the early stages of disease progression.

Three sets of DEGs (hsa-miR-22-3p, hsa-miR-129-1-3p, and hsa-miR-365a-3p) were subjected to functional enrichment analysis. The aforementioned three miRNAs have been studied in the context of tumors, including breast cancer and intestinal tumors, and for their protective role in chemotherapeutic drugs against myocardial toxicity. Li et al. (2020) constructed miRNA expression profiles of rutin (RUT) interfering with anthracycline pirarubicin (THP)-induced cardiotoxicity in rats using microarray technology. They found that RUT reversed these results, suggesting that miR-129-1-3p might be a new therapeutic target for THP-induced cardiotoxicity and breast cancer. Circonol C1 promotes breast cancer progression by targeting the miR-365a-3p/STAT3 axis, while propofol inhibits Circonol C1 by reducing STAT3 expression (Liu et al., 2021). Previous studies have found that miR-129-1-3p, subjected to cyclic stretch, can activate Runx2 and vascular endothelial growth factors (VEGFs) to promote endothelial differentiation and angiogenesis in endothelial progenitor cells (EPCs), which could be a potential candidate for treating vascular injury (Li et al., 2019). miR-22 has been well-studied in various tumor metastases. It is an important epigenetic regulator that promotes epithelial–mesenchymal transition (EMT) and multifunctional metastasis in breast cancer (Kong et al., 2014). During the last decade, miR-22 has been shown to participate in angiogenesis, age-associated vascular diseases, and cardiac hypertrophy (Gurha et al., 2012a; Zheng and Xu, 2014; Takeda et al., 2016). miR-22 is significantly elevated in the aging rat heart, which partly accelerates cardiac fibroblast senescence (Jazbutyte et al., 2012). Targeted miR-22 knockdown promotes myocardial contractile function dysregulation (Gurha et al., 2012b). In an ischemia–reperfusion injury model, miR-22 was found to protect the heart by directly targeting the CREB binding protein (CBP), in part, through the CBP/AP-1 pathway to reduce apoptosis and inflammatory injury (Yang et al., 2014). Another observational study analyzed patients with CAD, including stable angina, unstable angina, non-ST-segment elevation myocardial infarction, or ST-segment elevation myocardial infarction, and found significantly reduced miR-22 expression levels in PBMCs using a real-time polymerase chain reaction (qRT-PCR) assay (Chen et al., 2016). However, the results of some studies are consistent with our results. These studies found that circulating miR-22-3p was significantly upregulated in CAD patients compared to healthy subjects (Coffey et al., 2015; Zhong et al., 2020). Therefore, in patients with coronary heart disease, the increase and decrease in miRNA levels is affected by the sample sources (such as heart tissue, plasma, platelets, and monocytes) and complications.

Enrichment analysis was performed on 352 predicted target genes. The DEG-enriched pathways mainly included transcriptional misregulation, growth hormone synthesis, secretion and action, endocrine resistance, axon guidance, and Cushing syndrome. The Pathview-enriched pathways were mainly related to the Hippo signaling and prion disease pathways. The Hippo signaling pathway is a cellular signaling pathway that is significant in animal development and is responsible for regulating cell proliferation and organ growth (Zhao et al., 2019). The core of the mammalian Hippo signaling pathway consists of large tumor suppressor kinase 1/2 (LATS1/LATS2), macrophage stimulating 1/2 (MST1/MST2), tumor suppressor protein MOB1, and transcriptional activator protein yes-associated protein (YAP) (White et al., 2019). The crystal structure of the human MOB1-NDR2 protein complex has been analyzed to show that MOB1 binding to LATS1/2 is essential for tissue growth and organ development, whereas MOB1 binding to MST1/2 is not vital (Moya et al., 2019). Significant discoveries have been made in the study of the Hippo pathway in the cardiovascular system (Ardestani et al., 2018; Wang et al., 2018). YAP/TAZ is crucial in the proliferation and differentiation of progenitor cells and contributes to homeostasis maintenance in adult cardiomyocytes (Mosqueira et al., 2014). The Hippo-YAP pathway alters the production or degradation of the extracellular matrix and growth and migration of vascular smooth muscle cells and endothelial cells, thereby promoting vascular remodeling (He et al., 2018). CAD-associated functional proteins negatively regulate Hippo signaling in the endothelium, causing increased activity of YAP, a transcriptional effector of this pathway, leading to endothelial cell dysfunction that contributes to atherogenesis (Jones et al., 2018). YAP activation after myocardial infarction preserves cardiac function and reduces infarct size. Cardiac-specific YAP activation reduces myocardial injury, promotes myocardial regeneration and repair, improves cardiac function, and may improve survival (Lin et al., 2016). Functional enrichment analysis showed that miR-21-5p and miR-135b are associated with the Wnt and Hippo pathways, respectively, and may be associated with arrhythmogenic right ventricular cardiomyopathy (ARVC) (Byun et al., 2019). In conclusion, the Hippo signaling pathway is vital in cardiovascular processes, such as vascular smooth muscle cell remodeling, vascular endothelial growth, cardiac regenerative repair, and cardiomyopathy. However, its signaling interactions, pathophysiological mechanisms, and functional roles in the cardiovascular system remain to be investigated in depth.

We constructed an mRNA–miRNA–lincRNA ceRNA interaction network centered on miR-22-3p and used the starBase v2.0 and miRWalk databases to predict eight related transcription factors associated with cardiovascular disease, namely, CTCF, JUN, JUND, NFATC1, NFE2L2, RAD21, RELA, and TAL1. Several studies have been conducted on the aforementioned hub genes in various diseases. For instance, CTCF is a key chromatin architecture protein that binds to insulators, regulates enhancer–promoter interactions for transcriptional insulation, and acts as a transcriptional repressor to regulate gene expression (Shukla et al., 2011; Huang et al., 2021). More studies have shown that CTCF protein binding is influenced by DNA methylation levels in the cystathionine β-synthase (CBS) motif region and that CTCF haplotype dose expression can affect DNA methylation stability (Kemp et al., 2014). Nuclear factor E2-related factor 2 (NRF2) is encoded by the *NFE2L2* gene, and the NRF2/ARE signaling pathway is considered a potential therapeutic strategy for antioxidative stress-mediated diseases, such as diabetes, fibrosis, and cancer (Thiruvengadam et al., 2021). *NFE2L2* polymorphism is associated with acute type A aortic coarctation risk and severity in a Chinese Han population (Zhang et al., 2021). RAD21, RELA, and TAL1 are reportedly associated with tumors (Wu et al., 2019; Cao et al., 2021; Wang et al., 2021). However, few studies have investigated the relationship between hub genes and CAD. Therefore, this study is the first to potentiate eight CAD-associated hub genes (CTCF, JUN, JUND, NFATC1, NFE2L2, RAD21, RELA, and TAL1). However, this study had some limitations. First, the sample size for this study was relatively small, which could be a significant factor. Second, this project only completed bioinformatics analysis and did not provide experiments to further validate the aforementioned results. Subsequent studies will include cellular and animal experimental mechanism studies and clinical sample histological studies.

In conclusion, the present study suggests that miR-22-3p may be crucial in the onset and course of CAD. Our findings may provide potential targets for future CAD diagnoses and treatments.

## Data Availability

Publicly available datasets were analyzed in this study. These data can be found here: https://www.ncbi.nlm.nih.gov/geo/.

## Supplementary material

SUPPLEMENTARY FIGURE S1 Boxplot plots before and after sample correction for two datasets. **(A)** and **(B)** represent the boxplot plots before and after sample correction with inter-batch differences removed for the GSE105449 dataset, respectively. **(C)** and **(D)** represent the boxplot plots before and after sample correction with inter-batch differences removed for the GSE28858 dataset, respectively.

SUPPLEMENTARY FIGURE S2 PCA plots before and after sample correction for the two datasets. **(A)** and **(B)** represent the PCA plots before and after sample correction with inter-batch differences removed for the GSE105449 dataset, respectively. **(C)** and **(D)** represent the PCA plots before and after samples correction with inter-batch differences removed for the GSE28858 dataset, respectively.

## References

[B1] Ardestani A. Lupse B. Maedler K. (2018). Hippo signaling: Key emerging pathway in cellular and whole-body metabolism. Trends Endocrinol. Metab. 29 (7), 492–509. 10.1016/j.tem.2018.04.006 29739703

[B2] Byun J. Del Re D. Zhai P. Ikeda S. Shirakabe A. Mizushima W. (2019). Yes-associated protein (YAP) mediates adaptive cardiac hypertrophy in response to pressure overload. J. Biol. Chem. 294 (10), 3603–3617. 10.1074/jbc.RA118.006123 30635403 PMC6416448

[B3] Cao H. Wang D. Sun P. Chen L. Feng Y. Gao R. (2021). RNA-seq reveals microRNA-302b as a suppressor of prostate cancer epithelial-mesenchymal transition by targeting RELA/NF-κB. Am. J. Cancer Res. 11 (11), 5715–5725. 34873489 PMC8640823

[B4] Chen B. Luo L. Zhu W. Wei X. Li S. Huang Y. (2016). miR-22 contributes to the pathogenesis of patients with coronary artery disease by targeting MCP-1: An observational study. Medicine 95 (33), e4418. 10.1097/MD.0000000000004418 27537567 PMC5370794

[B5] Cheng S. Xie W. Miao Y. Guo J. Wang J. Li C. (2019). Identification of key genes in invasive clinically non-functioning pituitary adenoma by integrating analysis of DNA methylation and mRNA expression profiles. J. Transl. Med. 17 (1), 407. 10.1186/s12967-019-02148-3 31796052 PMC6892283

[B6] Coffey S. Williams M. J. A. Phillips L. V. Jones G. T. (2015). Circulating microRNA profiling needs further refinement before clinical use in patients with aortic stenosis. J. Am. Heart Assoc. 4 (8), e002150. 10.1161/JAHA.115.002150 26304936 PMC4599470

[B7] de Ronde M. W. J. Kok M. G. M. Moerland P. D. Van den Bossche J. Neele A. E. Halliani A. (2017). High miR-124-3p expression identifies smoking individuals susceptible to atherosclerosis. Atherosclerosis 263, 377–384. 10.1016/j.atherosclerosis.2017.03.045 28457624

[B8] Du L. Xu Z. Wang X. Liu F. (2020). Integrated bioinformatics analysis identifies microRNA-376a-3p as a new microRNA biomarker in patient with coronary artery disease. Am. J. Transl. Res. 12 (2), 633–648. 32194911 PMC7061823

[B9] Dweep H. Gretz N. (2015). miRWalk2.0: a comprehensive atlas of microRNA-target interactions. Nat. Methods 12 (8), 697. 10.1038/nmeth.3485 26226356

[B10] Gautier L. Cope L. Bolstad B. M. Irizarry R. A. (2004). affy--analysis of Affymetrix GeneChip data at the probe level. Bioinforma. Oxf. Engl. 20 (3), 307–315. 10.1093/bioinformatics/btg405 14960456

[B11] Gu Y. Ma X. Li J. Ma Y. Zhang Y. (2021). Identification of candidate targets for the diagnosis and treatment of atherosclerosis by bioinformatics analysis. Am. J. Transl. Res. 13 (5), 4137–4151. 34150004 PMC8205787

[B12] Gurha P. Abreu-Goodger C. Wang T. Ramirez M. O. Drumond A. L. van Dongen S. (2012a). Targeted deletion of microRNA-22 promotes stress-induced cardiac dilation and contractile dysfunction. Circulation 125 (22), 2751–2761. 10.1161/CIRCULATIONAHA.111.044354 22570371 PMC3503489

[B13] Gurha P. Abreu-Goodger C. Wang T. Ramirez M. O. Drumond A. L. van Dongen S. (2012b). Targeted deletion of MicroRNA-22 promotes stress-induced cardiac dilation and contractile dysfunction. Circulation 125 (22), 2751–2761. 10.1161/circulationaha.111.044354 22570371 PMC3503489

[B14] He J. Bao Q. Yan M. Liang J. Zhu Y. Wang C. (2018). The role of Hippo/yes-associated protein signalling in vascular remodelling associated with cardiovascular disease. Br. J. Pharmacol. 175 (8), 1354–1361. 10.1111/bph.13806 28369744 PMC5866970

[B15] Hu Y. Matkovich S. J. Hecker P. A. Zhang Y. Edwards J. R. Dorn G. W. (2012). Epitranscriptional orchestration of genetic reprogramming is an emergent property of stress-regulated cardiac microRNAs. Proc. Natl. Acad. Sci. U. S. A. 109 (48), 19864–19869. 10.1073/pnas.1214996109 23150554 PMC3511760

[B16] Huang H. Zhu Q. Jussila A. Han Y. Bintu B. Kern C. (2021). CTCF mediates dosage- and sequence-context-dependent transcriptional insulation by forming local chromatin domains. Nat. Genet. 53 (7), 1064–1074. 10.1038/s41588-021-00863-6 34002095 PMC8853952

[B17] Huang Z. P. Chen J. Seok H. Y. Zhang Z. Kataoka M. Hu X. (2013). MicroRNA-22 regulates cardiac hypertrophy and remodeling in response to stress. Circ. Res. 112 (9), 1234–1243. 10.1161/CIRCRESAHA.112.300682 23524588 PMC3720677

[B18] Jazbutyte V. Fiedler J. Kneitz S. Galuppo P. Just A. Holzmann A. (2012). MicroRNA-22 increases senescence and activates cardiac fibroblasts in the aging heart. Age 35 (3), 747–762. 10.1007/s11357-012-9407-9 22538858 PMC3636396

[B19] Jones P. D. Kaiser M. A. Ghaderi Najafabadi M. Koplev S. Zhao Y. Douglas G. (2018). JCAD, a gene at the 10p11 coronary artery disease locus, regulates Hippo signaling in endothelial cells. Arterioscler. Thromb. Vasc. Biol. 38 (8), 1711–1722. 10.1161/ATVBAHA.118.310976 29794114 PMC6296439

[B20] Karakas M. Schulte C. Appelbaum S. Ojeda F. Lackner K. Münzel T. (2017). Circulating microRNAs strongly predict cardiovascular death in patients with coronary artery disease-results from the large AtheroGene study. Eur. Heart J. 38 (7), 516–523. 10.1093/eurheartj/ehw250 27357355

[B21] Kemp C. J. Moore J. M. Moser R. Bernard B. Teater M. Smith L. E. (2014). CTCF haploinsufficiency destabilizes DNA methylation and predisposes to cancer. Cell Rep. 7 (4), 1020–1029. 10.1016/j.celrep.2014.04.004 24794443 PMC4040130

[B22] Kok M. Halliani A. Moerland P. Meijers J. Creemers E. Pinto-Sietsma S. (2015). Normalization panels for the reliable quantification of circulating microRNAs by RT-qPCR. FASEB J. official Publ. Fed. Am. Soc. Exp. Biol. 29 (9), 3853–3862. 10.1096/fj.15-271312 26023181

[B23] Kolde R. (2019). pheatmap: Pretty Heatmaps. R package. *version 1.0.12* . Available at: https://CRAN.R-project.org/package=pheatmap .

[B24] Kong L. M. Liao C. G. Zhang Y. Xu J. Li Y. Huang W. (2014). A regulatory loop involving miR-22, Sp1, and c-Myc modulates CD147 expression in breast cancer invasion and metastasis. Cancer Res. 74 (14), 3764–3778. 10.1158/0008-5472.CAN-13-3555 24906624

[B25] Li N. Wang W.-B. Bao H. Shi Q. Jiang Z.-L. Qi Y.-X. (2019). MicroRNA-129-1-3p regulates cyclic stretch-induced endothelial progenitor cell differentiation by targeting Runx2. J. Cell. Biochem. 120 (4), 5256–5267. 10.1002/jcb.27800 30320897

[B26] Li Q. Qin M. Tan Q. Li T. Gu Z. Huang P. (2020). MicroRNA-129-1-3p protects cardiomyocytes from pirarubicin-induced apoptosis by down-regulating the GRIN2D-mediated Ca2+ signalling pathway. J. Cell. Mol. Med. 24 (3), 2260–2271. 10.1111/jcmm.14908 31957170 PMC7011137

[B27] Lin J. Chen W. Gong M. Xu X. Du M. Wang S. (2021). Expression and functional analysis of lncRNAs involved in platelet-derived growth factor-BB-induced proliferation of human aortic smooth muscle cells. Front. Cardiovasc. Med. 8, 702718. 10.3389/fcvm.2021.702718 34557530 PMC8452921

[B28] Lin Z. Guo H. Cao Y. Zohrabian S. Zhou P. Ma Q. (2016). Acetylation of VGLL4 regulates hippo-YAP signaling and postnatal cardiac growth. Dev. Cell 39 (4), 466–479. 10.1016/j.devcel.2016.09.005 27720608 PMC5121000

[B29] Liu Y. Heng J. Zhao X. Li E. (2021). The inhibition of circular RNA circNOLC1 by propofol/STAT3 attenuates breast cancer stem cells function via miR-365a-3p/STAT3 signaling. J. Transl. Med. 19 (1), 467. 10.1186/s12967-021-03133-5 34789263 PMC8596799

[B30] Ma L. Li A. Zou D. Xu X. Xia L. Yu J. (2015). LncRNAWiki: Harnessing community knowledge in collaborative curation of human long non-coding RNAs. Nucleic Acids Res. 43, D187–D192. 10.1093/nar/gku1167 25399417 PMC4383965

[B31] Mayr B. Müller E. Schäfer C. Droese S. Schönfelder M. Niebauer J. (2021). Exercise-induced changes in miRNA expression in coronary artery disease. Clin. Chem. Lab. Med. 59, 1719–1727. 10.1515/cclm-2021-0164 33977686

[B32] Meng Y. Zhang C. Liang L. Wei L. Wang H. Zhou F. (2021). Identification of potential key genes involved in the carotid atherosclerosis. Clin. Interv. Aging 16, 1071–1084. 10.2147/cia.s312941 34140767 PMC8203271

[B33] Mosqueira D. Pagliari S. Uto K. Ebara M. Romanazzo S. Escobedo-Lucea C. (2014). Hippo pathway effectors control cardiac progenitor cell fate by acting as dynamic sensors of substrate mechanics and nanostructure. ACS Nano 8 (3), 2033–2047. 10.1021/nn4058984 24483337

[B34] Moya I. M. Castaldo S. A. Van den Mooter L. Soheily S. Sansores-Garcia L. Jacobs J. (2019). Peritumoral activation of the Hippo pathway effectors YAP and TAZ suppresses liver cancer in mice. Sci. (New York, N.Y.) 366 (6468), 1029–1034. 10.1126/science.aaw9886 31754005

[B35] Nariman-Saleh-Fam Z. Vahed S. Aghaee-Bakhtiari S. Daraei A. Saadatian Z. Kafil H. (2019). Expression pattern of miR-21, miR-25 and PTEN in peripheral blood mononuclear cells of patients with significant or insignificant coronary stenosis. Gene 698, 170–178. 10.1016/j.gene.2019.02.074 30849539

[B36] Pandey D. Picard D. (2009). miR-22 inhibits estrogen signaling by directly targeting the estrogen receptor alpha mRNA. Mol. Cell. Biol. 29 (13), 3783–3790. 10.1128/mcb.01875-08 19414598 PMC2698751

[B37] Raggi P. Genest J. Giles J. T. Rayner K. J. Dwivedi G. Beanlands R. S. (2018). Role of inflammation in the pathogenesis of atherosclerosis and therapeutic interventions. Atherosclerosis 276, 98–108. 10.1016/j.atherosclerosis.2018.07.014 30055326

[B38] Ritchie M. E. Phipson B. Wu D. Hu Y. Law C. W. Shi W. (2015). Limma powers differential expression analyses for RNA-sequencing and microarray studies. Nucleic Acids Res. 43 (7), e47. 10.1093/nar/gkv007 25605792 PMC4402510

[B39] Saadatian Z. Nariman-Saleh-Fam Z. Bastami M. Mansoori Y. Khaheshi I. Parsa S. (2019). Dysregulated expression of STAT1, miR-150, and miR-223 in peripheral blood mononuclear cells of coronary artery disease patients with significant or insignificant stenosis. J. Cell. Biochem. 120 (12), 19810–19824. 10.1002/jcb.29286 31318097

[B40] Shukla S. Kavak E. Gregory M. Imashimizu M. Shutinoski B. Kashlev M. (2011). CTCF-promoted RNA polymerase II pausing links DNA methylation to splicing. Nature 479 (7371), 74–79. 10.1038/nature10442 21964334 PMC7398428

[B41] Sondermeijer B. M. Bakker A. Halliani A. de Ronde M. W. Marquart A. A. Tijsen A. J. (2011). Platelets in patients with premature coronary artery disease exhibit upregulation of miRNA340* and miRNA624. PLoS One 6 (10), e25946. 10.1371/journal.pone.0025946 22022480 PMC3192762

[B42] Su M. Niu Y. Dang Q. Qu J. Zhu D. Tang Z. (2020). Circulating microRNA profiles based on direct S-Poly(T)Plus assay for detection of coronary heart disease. J. Cell. Mol. Med. 24 (11), 5984–5997. 10.1111/jcmm.15001 32343493 PMC7294166

[B43] Takeda E. Suzuki Y. Sato Y. (2016). Age-associated downregulation of vasohibin-1 in vascular endothelial cells. Aging Cell 15 (5), 885–892. 10.1111/acel.12497 27325558 PMC5013028

[B44] Tan X. Zhang X. Pan L. Tian X. Dong P. (2017). Identification of key pathways and genes in advanced coronary atherosclerosis using bioinformatics analysis. Biomed. Res. Int. 2017, 4323496. 10.1155/2017/4323496 29226137 PMC5684517

[B45] Tanase D. M. Gosav E. M. Ouatu A. Badescu M. C. Dima N. Ganceanu-Rusu A. R. (2021). Current knowledge of MicroRNAs (miRNAs) in acute coronary syndrome (ACS): ST-elevation myocardial infarction (STEMI). Life (Basel, Switz. 11 (10), 1057. 10.3390/life11101057 PMC8541211 34685428

[B46] Thiruvengadam M. Venkidasamy B. Subramanian U. Samynathan R. Ali Shariati M. Rebezov M. (2021). Bioactive compounds in oxidative stress-mediated diseases: Targeting the NRF2/ARE signaling pathway and epigenetic regulation. Antioxidants (Basel, Switz. 10 (12), 1859. 10.3390/antiox10121859 PMC8698417 34942962

[B47] Thomas M. R. Lip G. Y. H. (2017). Novel risk markers and risk assessments for cardiovascular disease. Circ. Res. 120 (1), 133–149. 10.1161/CIRCRESAHA.116.309955 28057790

[B48] Vlachos I. S. Zagganas K. Paraskevopoulou M. D. Georgakilas G. Karagkouni D. Vergoulis T. (2015). DIANA-miRPath v3.0: Deciphering microRNA function with experimental support. Nucleic Acids Res. 43 (W1), W460–W466. 10.1093/nar/gkv403 25977294 PMC4489228

[B49] Wang J. Liu S. Heallen T. Martin J. F. (2018). The Hippo pathway in the heart: Pivotal roles in development, disease, and regeneration. Nat. Rev. Cardiol. 15 (11), 672–684. 10.1038/s41569-018-0063-3 30111784

[B50] Wang J. Zhao H. Yu J. Xu X. Jing H. Li N. (2021). MiR-320b/RAD21 axis affects hepatocellular carcinoma radiosensitivity to ionizing radiation treatment through DNA damage repair signaling. Cancer Sci. 112 (2), 575–588. 10.1111/cas.14751 33251678 PMC7894001

[B51] White S. Murakami S. Yi C. (2019). The complex entanglement of Hippo-Yap/Taz signaling in tumor immunity. Oncogene 38 (16), 2899–2909. 10.1038/s41388-018-0649-6 30617303 PMC7567008

[B52] Wilkinson L. (2011). ggplot2: Elegant graphics for data analysis by WICKHAM, H. Biometrics 67 (2), 678–679. 10.1111/j.1541-0420.2011.01616.x

[B53] Wu Y. Hu Y. Yu X. Zhang Y. Huang X. Chen S. (2019). TAL1 mediates imatinib-induced CML cell apoptosis via the PTEN/PI3K/AKT pathway. Biochem. Biophys. Res. Commun. 519 (2), 234–239. 10.1016/j.bbrc.2019.08.164 31493871

[B54] Yang J. Chen L. Yang J. Ding J. Li S. Wu H. (2014). MicroRNA-22 targeting CBP protects against myocardial ischemia-reperfusion injury through anti-apoptosis in rats. Mol. Biol. Rep. 41 (1), 555–561. 10.1007/s11033-013-2891-x 24338162

[B55] Yu G. Wang L.-G. Han Y. He Q.-Y. (2012). clusterProfiler: an R package for comparing biological themes among gene clusters. Omics a J. Integr. Biol. 16 (5), 284–287. 10.1089/omi.2011.0118 PMC3339379 22455463

[B56] Zeng Z. Xia L. Fan S. Zheng J. Qin J. Fan X. (2021). Circular RNA CircMAP3K5 acts as a MicroRNA-22-3p sponge to promote resolution of intimal hyperplasia via TET2-mediated smooth muscle cell differentiation. Circulation 143 (4), 354–371. 10.1161/circulationaha.120.049715 33207953

[B57] Zhang M. Li Y. Xie H. Chen J. Liu S. (2020). Curcumin inhibits proliferation, migration and neointimal formation of vascular smooth muscle via activating miR-22. Pharm. Biol. 58 (1), 610–619. 10.1080/13880209.2020.1781904 32631202 PMC8641690

[B58] Zhang Y. Zheng Q. Chen R. Dai X. Zhu Y. Ma L. (2021). Association of NFE2L2 gene polymorphisms with risk and clinical characteristics of acute type A aortic dissection in han Chinese population. Oxid. Med. Cell. Longev. 2021, 5173190. 10.1155/2021/5173190 34336095 PMC8313362

[B59] Zhao W. Lu Q. Nguyen M. Su Y. Ziemann M. Wang L. (2019). Stimulation of β-adrenoceptors up-regulates cardiac expression of galectin-3 and BIM through the Hippo signalling pathway. Br. J. Pharmacol. 176 (14), 2465–2481. 10.1111/bph.14674 30932177 PMC6592853

[B60] Zhelankin A. Stonogina D. Vasiliev S. Babalyan K. Sharova E. Doludin Y. (2021). Circulating extracellular miRNA analysis in patients with stable CAD and acute coronary syndromes. Biomolecules 11 (7), 962. 10.3390/biom11070962 34209965 PMC8301961

[B61] Zheng Y. Xu Z. (2014). MicroRNA-22 induces endothelial progenitor cell senescence by targeting AKT3. Cell. Physiol. biochem. 34 (5), 1547–1555. 10.1159/000366358 25323119

[B62] Zhong Z. Zhong W. Zhang Q. Zhang Q. Yu Z. Wu H. (2020). Circulating microRNA expression profiling and bioinformatics analysis of patients with coronary artery disease by RNA sequencing. J. Clin. Lab. Anal. 34 (1), e23020. 10.1002/jcla.23020 31489700 PMC6977390

