# Supplementary material for: miR-22-3p as a potential biomarker for coronary artery disease based on integrated bioinformatics analysis
Source: Front Genet. 2022 Aug 29;13:936937. doi: 10.3389/fgene.2022.936937 (PMC9464939; doi:10.3389/fgene.2022.936937)
Supplement: Supplementary file 3 [file Datasheet1.pdf]

Table1: Significantly up-regulated or down-regulated miRNAs in CAD in comparison to controls.

	logFC	AveExpr	t	*P*.Value
hsa-miR-1274b_v16.0	-0.60545	10.56429	-2.37852	0.02253
hsa-miR-1305	-0.38721	5.004401	-2.17605	0.035859
hsa-miR-720	-0.30556	12.70364	-2.08204	0.044147
hsa-miR-365a-3p	-0.26083	8.952461	-2.74107	0.009293
hsa-miR-1288	-0.24668	3.805899	-2.07349	0.044979
hsa-miR-892b	-0.1729	3.581098	-2.24984	0.030351
hsa-miR-129-1-3p	-0.15594	3.474189	-2.0747	0.044861
hsa-miR-505-3p	0.196021	6.605712	2.377145	0.022604
hsa-miR-22-3p	0.208511	11.97119	2.430924	0.019905
